# Supplementary material for: Sleeping sickness is a circadian disorder
Source: Nat Commun. 2018 Jan 4;9:62. doi: 10.1038/s41467-017-02484-2 (PMC5754353; doi:10.1038/s41467-017-02484-2)
Supplement: Supplementary file 1 — Supplementary Information [file 41467_2017_2484_MOESM1_ESM.pdf]

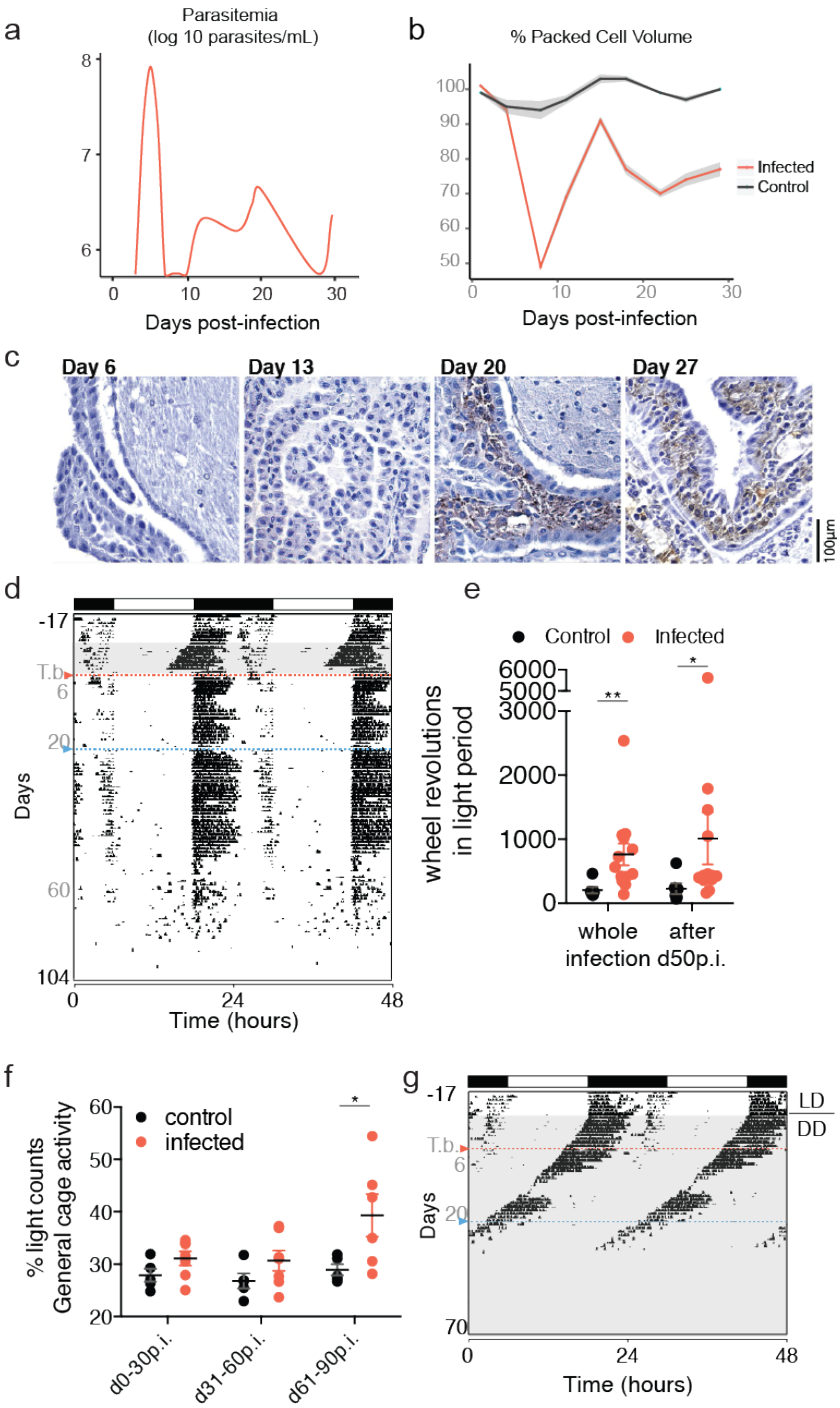
a
Parasitemia
(log 10 parasites/mL)
8
7
6
0
10
20
30
Days post-infection
b
% Packed Cell Volume
100
90
80
70
60
50
0
10
20
30
Infected
Control
Days post-infection
c
Day 6
Day 13
Day 20
Day 27
100µm
d
-17
T.b.
6
20
60
104
Days
0
24
48
Time (hours)
e
Control
Infected
wheel revolutions
in light period
6000
5000
3000
2000
1000
0
**
*
whole
infection
after
d50p.i.
f
% light counts
General cage activity
60
50
40
30
20
control
infected
*
d0-30p.i.
d31-60p.i.
d61-90p.i.
g
-17
T.b.
6
20
70
Days
LD
DD
0
24
48
Time (hours)

Supplementary Figure 1. Circadian activity and body temperature disruption in Trypanosoma-infected mice. a, Parasitemia profile of *T. brucei* infected mice. n = 5 mice **b**, Hematocrit of both control (black, n = 3) or Trypanosoma-infected (orange, n = 5) mice. Results represent average variation to baseline (day 0, day of injection) from each mouse. p = 0.0012, Two-Way ANOVA. **c**, Temporal histological analysis of infected brain. Representative microphotographs of the choroid plexuses (asterisk, in the lateral ventricles) (n = 5/time point), the area of the brain described as the parasite entry site into the central nervous system. Arrow indicates anti-VSG-stained *T. brucei* parasites. DAB counterstained with hematoxylin; original magnification, 40x. **d**, An additional actogram of daily wheel-running activity of infected mouse in light-dark cycles. Animals were treated with suramin (20 mg/kg) i.p. on day 21 post-infection. **e**, Total activity levels during rest period of mice infected and treated with suramin (20 mg/kg) on day 21. Error bars show mean ± SEM. *p < 0.05, **p < 0.01 by Mann-Whitney test. **f**, Telemetry measured general cage activity of control (n = 5) and infected (n = 7). Error bars show mean ± SEM. *p < 0.05 by unpaired t test. **g**, Additional actogram of daily wheel-running activity of infected mice in DD.

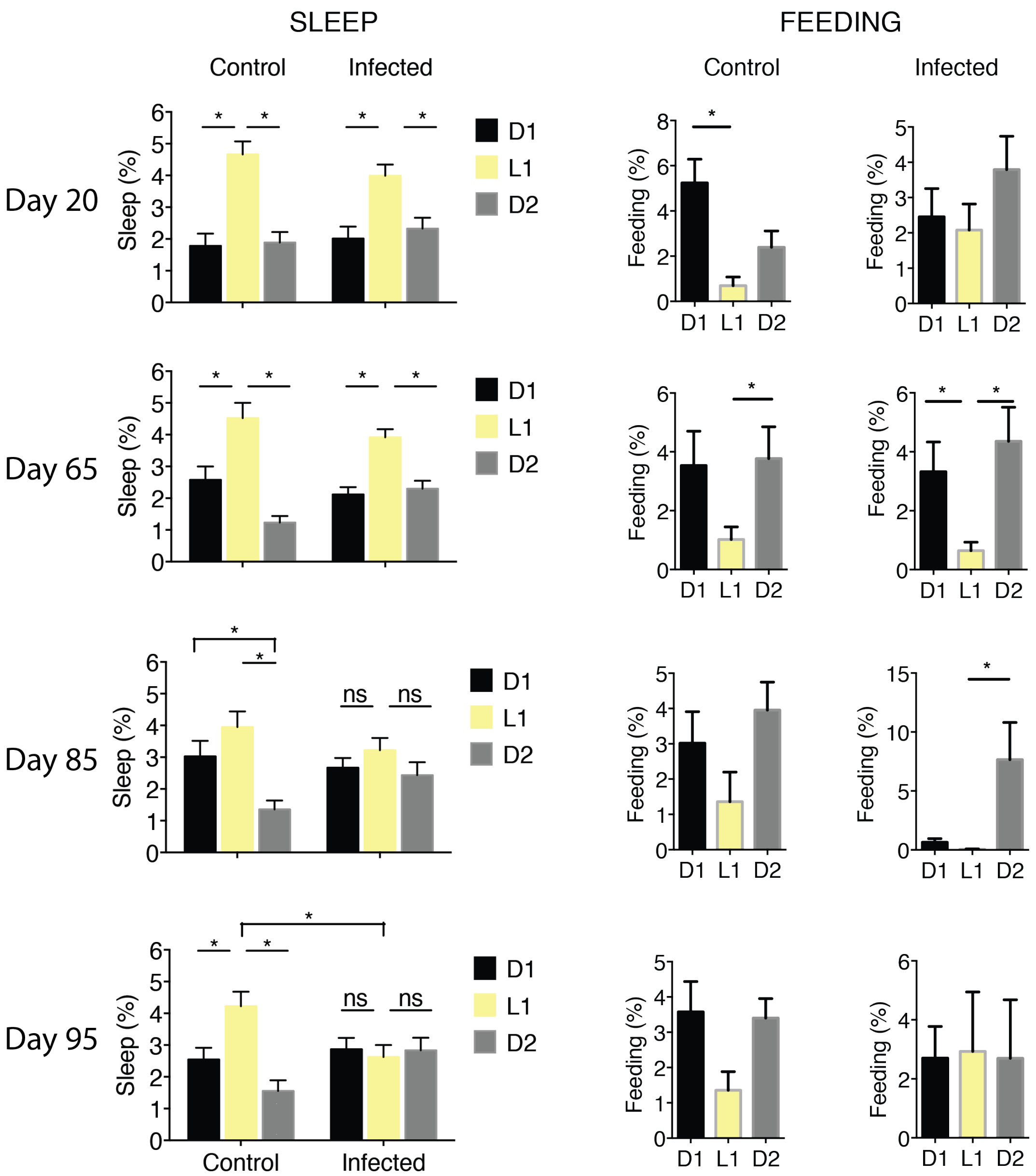

Supplementary Figure 2. Automated video analysis revealed sleep-like behavior disruption in *Trypanosoma brucei*-infected mice. Daily amount of time spent resting (sleep-like behavior) and feeding in both control and Trypanosoma-infected mice on days 20, 65, 85 and 95 post-infection. D1 represents the first night (12h of dark) and L1 the following daytime (12h of light) followed by a second night of recording (D2). Error bars represent SEM. n = 4-6 mice for controls and n = 3-5 mice in the infected group. * represents significance of two-way ANOVA for sleep and Friedman test for feeding.

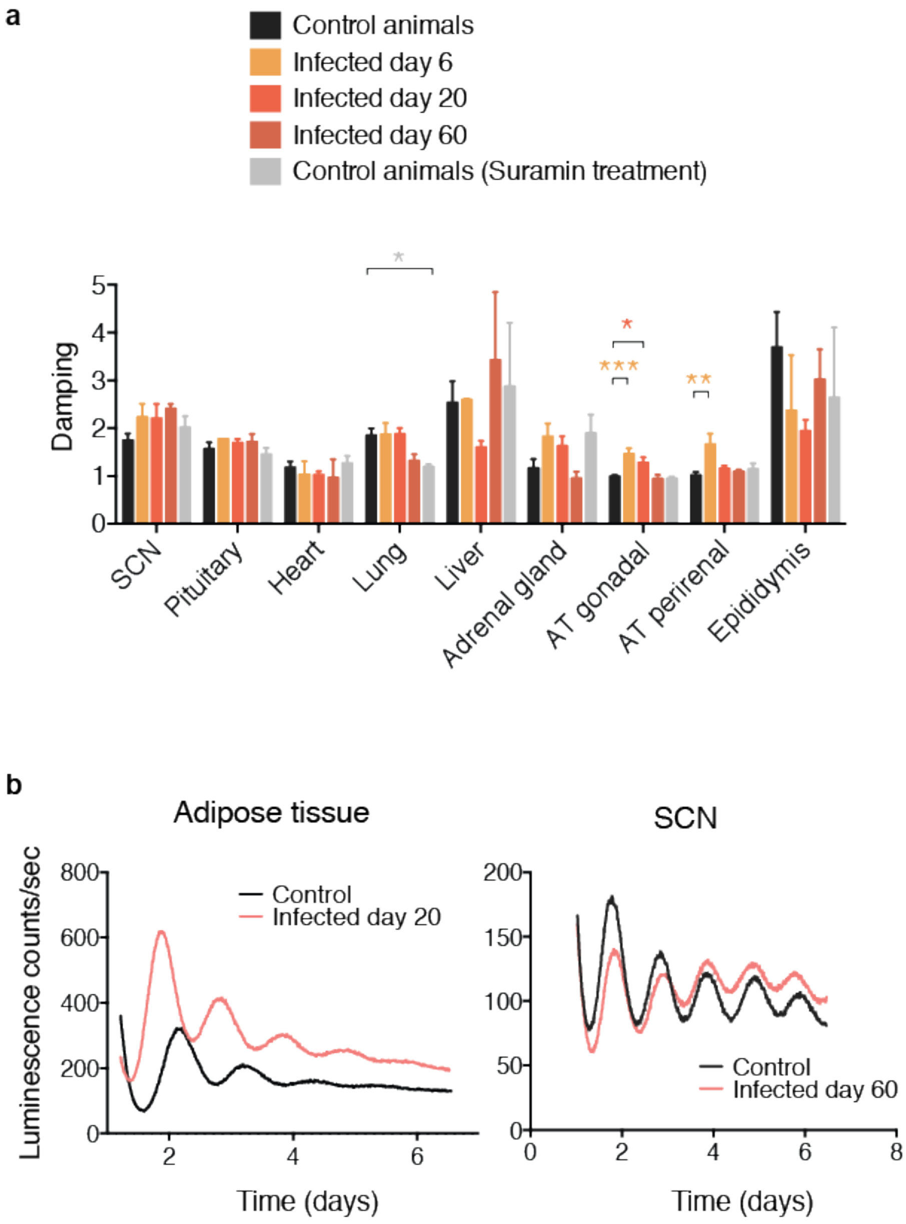

Supplementary Figure 3. Shorter period of PER2::LUC explants when high number of parasites is present. a, Damping analysis of various tissues harvested from control or infected mice in LD. Control (black, pooled from day 6 and day 20), infected day 6 (yellow), infected day 20 (orange), infected day 60 (dark orange, treated with suramin (20 mg/kg) on day 21) and control on day 60 treated with suramin (20 mg/kg) (gray). Shown are mean period ± SD. *$p < 0.05$, **$p < 0.01$, ***$p < 0.001$ by unpaired t test. **b**, Representative records of bioluminescence reporting of circadian expression from gonadal adipose tissue, day 20 and SCN on day 60 post-infection/vehicle.

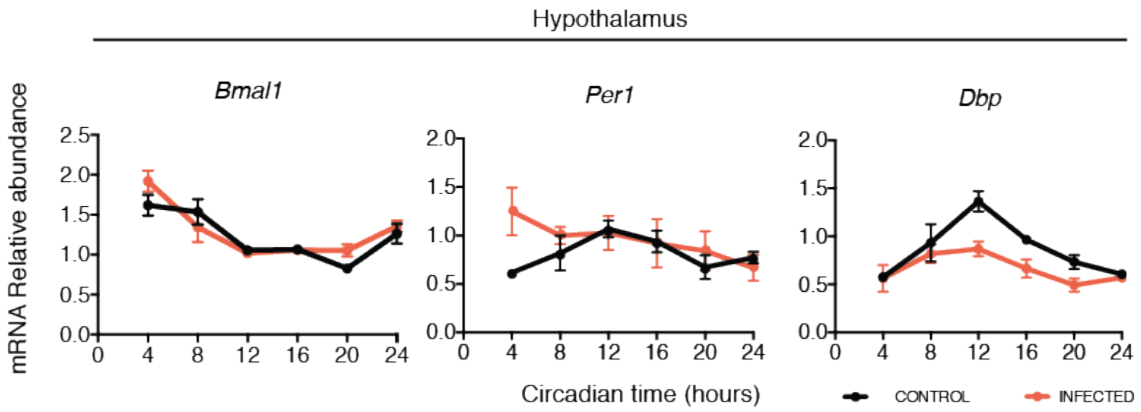

Supplementary Figure 4. Clock gene expression in the hypothalamus region. Real-time qPCR analysis of clock gene expression in control (black) and Trypanosoma-infected (orange) mice hypothalamus, area that includes the SCN – master clock. Error bars represent SEM for each time point from three independent replicates. Two-way ANOVA, $p > 0.05$ for both *Bmal1* and *Per1* mRNAs and $p = 0.0054$ for *Dbp* mRNA level comparison.

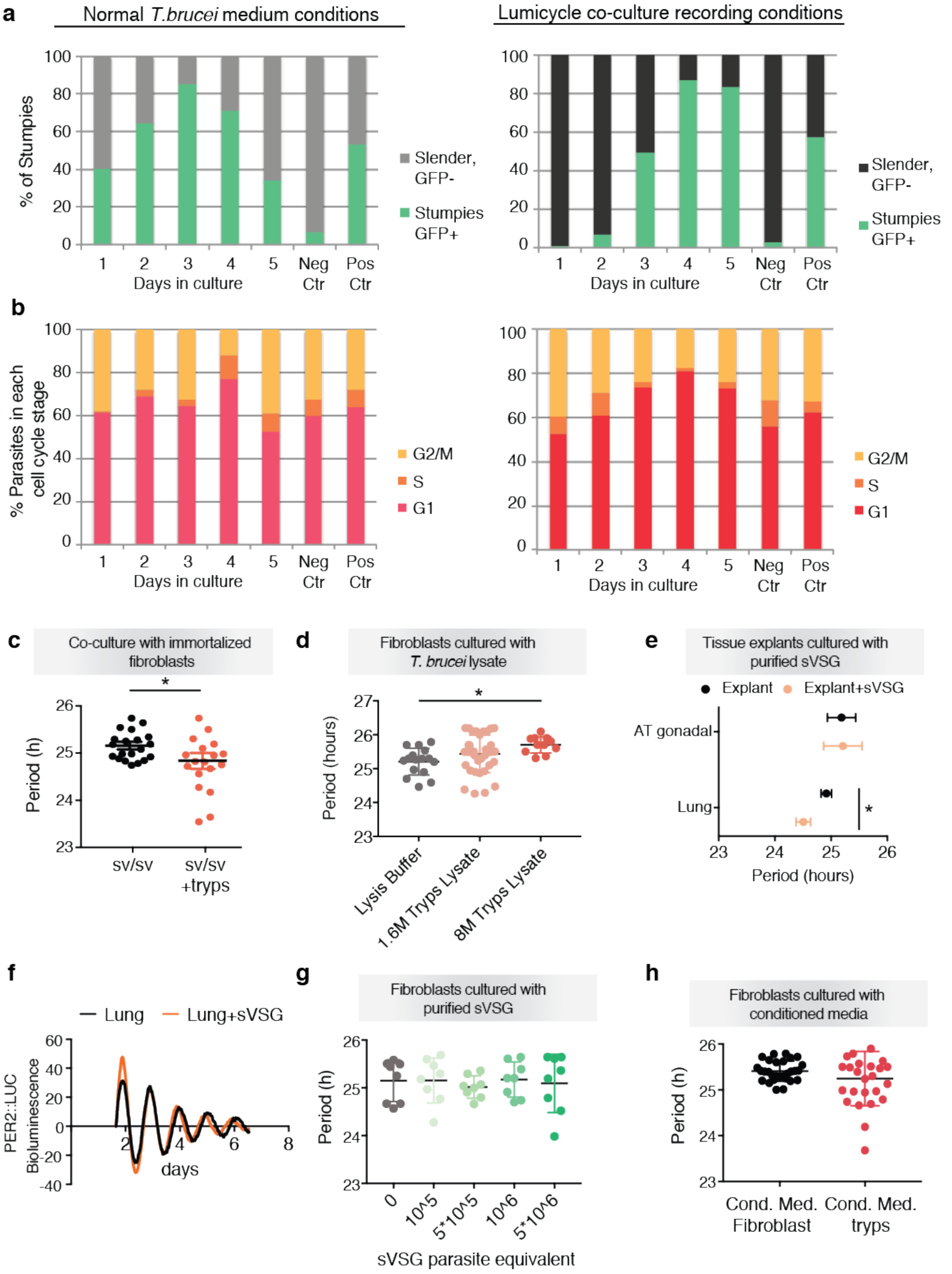
a
Normal T.brucei medium conditions
Lumicycle co-culture recording conditions
% of Stumpies
Slender, GFP-
Stumpies GFP+
Days in culture
Neg Ctr
Pos Ctr
b
% Parasites in each cell cycle stage
G2/M
S
G1
c
Co-culture with immortalized fibroblasts
Period (h)
sv/sv
sv/sv +tryps
d
Fibroblasts cultured with T. brucei lysate
Period (hours)
Lysis Buffer
1.6M Tryps Lysate
8M Tryps Lysate
e
Tissue explants cultured with purified sVSG
Explant
Explant+sVSG
AT gonadal
Lung
Period (hours)
f
Lung
Lung+sVSG
PER2::LUC Bioluminescence
days
g
Fibroblasts cultured with purified sVSG
Period (h)
0
10^5
5*10^5
10^6
5*10^6
sVSG parasite equivalent
h
Fibroblasts cultured with conditioned media
Period (h)
Cond. Med. Fibroblast
Cond. Med. tryps

Supplementary Figure 5. Co-culturing *T. brucei* with fibroblasts. (**a-b**) GFP::PAD1utr reporter *T. brucei* cell line was culture in normal parasite conditions (HMI11 and 5%CO_2) and co-culture luminescence recording conditions (TbM50 and air tight) for five days, starting at the $5x10^4$/mL density as in co-culture experiments (please see Material and Methods). Negative control is a culture of the parental *T. brucei* cell line AnTat 90:13 and positive control is GFP::PAD1utr reporter *T. brucei* cell line in HMI11 overgrown for 48h (i.e. 48h after reaching 10^6/mL). n = 5 independent cultures (biological replicates) in each of the five days of culture and in both control cultures. **c**, Period of PER2::LUC sv/sv fibroblasts. $p < 0.05$ Mann-Whitney test. n = 21 and 19 biological replicates for fibroblasts and fibroblasts co-cultured with *T. brucei*, respectively, from two independent experiments. **d**, PER2::LUC sv/sv fibroblasts cultured with lysates from 8 million or 1.6 million trypanosomes or just the lysis buffer (phosphate buffer with protease inhibitors). n = 14, 36 and 16 for lysates of 8, 1.6, 0 million trypanosomes, respectively, from three independent experiments. **e**, Tissue explants cultured with soluble VSG purified from 10^9 trypanosome cultures. n=10 per condition from four independent experiments. **f**, Luminescence profile of a lung explant with and without sVSG. **g**, Fibroblasts cultured with soluble VSG purified from trypanosome cultures. The serial dilutions reflect the equivalent of sVSG from 10^5 to $3x10^6$ trypanosomes. n = 8 per condition, from two independent experiments. **h,** PER2::LUC sv/sv fibroblasts cultured with 60% of conditioned media (CM) from parasites or fibroblasts and 40% fresh TbM50 media. n = 29 and 26 for fibroblasts CM and *T. brucei* CM, respectively, from three independent experiments.

Supplementary Table 1. Primer sequences used for gene expression quantification by qPCR

Gene	Fw/Rev	Seq (5' - 3')
Period 1	Fw	CCC AGC TTT ACC TGC AGA AG
Period 1	Rev	ATG GTC GAA AGG AAG CCT CT
Dbp	Fw	CGAAGAACGTCATGATGCAG
Dbp	Rev	GGTTCCCCAACATGCTAAGA
Gapdh	Fw	CAAGGAGTAAGAAACCCTGGACC
Gapdh	Rev	CGAGTTGGGATAGGGCCTCT
Bmal1	Fw	CCACCTCAGAGCCATTGATACA
Bmal1	Rev	GAGCAGGTTTAGTTCCACTTTGTCT
IL-10	Fw	TGCTATGCTGCCTGCTCTTA
IL-10	Rev	TCATTTCCGATAAGGCTTGG
TNF	Fw	AATGGCCTCCCTCTCATCAGTT
TNF	Rev	CCACTTGGTGGTTTGCTACGA
IL-1	Fw	GCCCATCCTCTGTGACTCAT
IL-1	Rev	AGGCCACAGGTATTTTGTCG
